# Mesenchymal Stromal Cell-Based Bone Regeneration Therapies: From Cell Transplantation and Tissue Engineering to Therapeutic Secretomes and Extracellular Vesicles

**DOI:** 10.3389/fbioe.2019.00352

**Published:** 2019-11-27

**Authors:** Darja Marolt Presen, Andreas Traweger, Mario Gimona, Heinz Redl

**Affiliations:** ^1^Ludwig Boltzmann Institute for Experimental and Clinical Traumatology, AUVA Research Center, Vienna, Austria; ^2^Austrian Cluster for Tissue Regeneration, Vienna, Austria; ^3^Spinal Cord Injury & Tissue Regeneration Center Salzburg, Institute of Tendon and Bone Regeneration, Paracelsus Medical University, Salzburg, Austria; ^4^GMP Unit, Spinal Cord Injury & Tissue Regeneration Center Salzburg, Paracelsus Medical University, Salzburg, Austria

**Keywords:** mesenchymal stromal cells, stem cells, MSCs, secretome, extracellular vesicles, cell therapy, bone tissue engineering, bone regeneration

## Abstract

Effective regeneration of bone defects often presents significant challenges, particularly in patients with decreased tissue regeneration capacity due to extensive trauma, disease, and/or advanced age. A number of studies have focused on enhancing bone regeneration by applying mesenchymal stromal cells (MSCs) or MSC-based bone tissue engineering strategies. However, translation of these approaches from basic research findings to clinical use has been hampered by the limited understanding of MSC therapeutic actions and complexities, as well as costs related to the manufacturing, regulatory approval, and clinical use of living cells and engineered tissues. More recently, a shift from the view of MSCs directly contributing to tissue regeneration toward appreciating MSCs as “cell factories” that secrete a variety of bioactive molecules and extracellular vesicles with trophic and immunomodulatory activities has steered research into new MSC-based, “cell-free” therapeutic modalities. The current review recapitulates recent developments, challenges, and future perspectives of these various MSC-based bone tissue engineering and regeneration strategies.

## Introduction

Regeneration of bone defects often presents significant challenges, particularly in patients with decreased tissue regeneration capacity due to extensive tissue damage, disease, advanced age, and confounding systemic and lifestyle factors (Gruber et al., 2006; Borrelli et al., 2012). Between 5 and 10% of all bone fractures, according to some reports even up to 50%, result in delayed or failed healing (Tzioupis and Giannoudis, 2007; Gómez-Barrena et al., 2015; Ekegren et al., 2018). To overcome these barriers, a number of research studies focused on enhancing bone regeneration by applying mesenchymal stromal cells (MSCs) derived from various connective tissues. *Ex vivo* processing and culture methods have been developed to obtain sufficient MSC numbers for therapy (Schallmoser et al., 2009; Sensebé et al., 2013; Robey et al., 2015). Furthermore, MSCs have been combined with various scaffolds and signaling factors in order to tissue engineer viable “bone substitutes” recapitulating key features of autologous bone grafts and enhancing bone regeneration (Frohlich et al., 2008; Jakob et al., 2012). *In vitro* culture of these constructs in order to drive cell differentiation, bone-like matrix deposition, and increased mechanical properties has also been extensively studied (Marolt et al., 2006; Grayson et al., 2011; Bhumiratana et al., 2016; Vetsch et al., 2016; Mitra et al., 2017; Zhao et al., 2018). Recapitulation of mechanisms present during embryonic bone development was proposed as a “developmental (re)engineering” strategy for the preparation of intermediate grafts capable of forming fully functional bone (Jukes et al., 2008; Tonnarelli et al., 2014; Bernhard et al., 2017). Viable, large bone-like grafts in clinically relevant dimensions (several millimeters to centimeters in size) have been achieved using dynamic culture of scaffolds seeded with MSCs in bioreactors (Grayson et al., 2010, 2011; Güven et al., 2011; Sørensen et al., 2012; Bhumiratana et al., 2016). In addition, in some cases these grafts comprised rudimentary vascular networks. Bone marrow and adipose tissue MSCs were used in the majority of preclinical and clinical studies (Marolt et al., 2010; Robey, 2011; Grayson et al., 2015; Nancarrow-Lei et al., 2017) (Table 1). However, various other sources of MSCs have also been investigated, including skeletal muscle, bone, cartilage, tendon, dental pulp, perinatal tissues (e.g., Wharton's Jelly, umbilical vein/cord blood, amnion, placenta), embryonic stem cells and induced pluripotent stem cells. Due to the aging-related decline in tissue regeneration (Kassem and Marie, 2011; Marie, 2014; Baker et al., 2015; Bhattacharjee et al., 2019), involving both intra- as well as extra-cellular mechanisms, perinatal tissues and induced pluripotent stem cells have raised interest as potential sources of “young” MSCs with high regenerative properties (Kern et al., 2006; Baksh et al., 2007; Robey, 2011; De Peppo et al., 2013; Ghasemzadeh et al., 2018; Spitzhorn et al., 2019).

**Table 1:** Clinical studies using MSCs and isolated progenitors for bone regeneration.

**Identifier**	**Phase**	**Completion**	**Condition**	**Cell type**	**Patients**	**Treatment groups**	**Masking**	**Main outcome (Reference)**
**SEARCH TERMS: “BONE FRACTURE, STEM CELLS”**								
NCT03325504	III	Recruiting	Non-union	Autologous bone marrow MSCs (*in vitro* expanded)	Est. 108	Low dose stem cell application with biomaterial High dose stem cell application with biomaterial Control autologous bone graft	None	/
NCT02483364	II	Recruiting	Pseudoarthrosis	Autologous or allogeneic adipose MSCs	Est. 12	Allogeneic stem cell application with tricalcium phosphate Autologous stem cell application with tricalcium phosphate	None	/
NCT02815423	I/II	Not yet recruiting	Non-union	Umbilical cord MSCs	Est. 40	Stem cell injection Control placebo injection	None	/
NCT01842477	I/II	February 2016	Delayed union Non-union	Autologous bone marrow MSCs (cultured)	30	Application of stem cells with bone substitute	None	No severe adverse events and 26/28 treated patients radiologically healed at 1 year (Gómez-Barrena et al., 2019)
NCT01813188	II	December 2013	Pseudoarthrosis	Autologous bone marrow MNCs	5	Application of cells seeded on tricalcium phosphate	None	/
NCT01788059	II	November 2013	Non-union	Autologous bone marrow MSCs (Ficoll separated)	19	Stem cell injection	None	/
NCT01581892	I/II	January 2013	Non-union	Autologous bone marrow MNCs (Ficoll separated)	7	Stem cell injection	None	/
NCT02177565	NA	October 2011	Non-union	Autologous bone marrow MSCs (*in vitro* expanded)	35	Stem cell application with carrier Control carrier alone	Double	/
NCT01206179	I	March 2011	Non-union	Autologous bone marrow MSCs (*in vitro* expanded)	6	Stem cell injection	None	Stem cell injections were tolerated with evidence of union in 3/5 patients (Emadedin et al., 2017)
NCT00916981	I/II	June 2009	Non-union Pseudoarthrosis	Autologous bone marrow derived pre-osteoblastic cells	30	Pre-osteoblastic cell injection	None	/
NCT02140528	II	April 2016	Tibial fracture	Allogeneic adipose MSCs	40	Stem cell injection Control placebo injection	Double	/
NCT00512434	NA	September 2013	Tibial fracture, open fracture	Autologous bone marrow MNCs	85	Stem cell injection and osteosynthesis Control osteosynthesis only	None	/
NCT00250302	I/II	April 2011	Tibial fracture	Autologous bone marrow MSCs (isolated)	24	Stem cell implantation with autologous platelet rich plasma/demineralized bone carrier Control no treatment	None	Shorter time to union in stem cell group (1.5 months) compared to control group (3 months) (Liebergall et al., 2013)
NCT02755922	III	December 2010	Mandibular fracture	Autologous adipose MSCs (24 h post-isolation)	20	Stem cell application Control no application	Single	Ossification values in stem cell group were similar to control at 4 weeks and higher as control at 12 weeks (Castillo-Cardiel et al., 2017)
NCT01532076	III	September 2014 (terminated)	Osteoporotic fracture	Autologous stromal vascular fraction	8	Application of cell-seeded hydroxyapatite/fibrin gel graft Control acellular composite graft	Single	/
**SEARCH TERMS: “BONE, STEM CELLS, MAXILLA, ALVEOLAR”**								
NCT03766217	III	Not yet recruiting	Cleft lip and palate	Autologous MSCs from deciduous dental pulp (enzyme isolated)	Est. 62	Application of stem cells with collagen and hydroxyapatite Control autologous bone graft	None	/
NCT02751125	I	Recruiting by invitation	Bone atrophy	Autologous bone marrow MSCs (cultured)	13	Application of stem cells mixed with biphasic calcium phosphate	None	Treatment resulted in bone formation sufficient for dental implant placement after 4–6 months (Gjerde et al., 2018)
NCT03070275	I/II	December 2017	Implant therapy	Autologous alveolar bone marrow MSCs (*in vitro* expanded)	20	Application of stem cells with autologous fibrin glue in collagen scaffold	Single	/
NCT02449005	I/II	December 2016	Chronic periodontitis	Autologous alveolar bone marrow MSCs (*in vitro* expanded)	30	Application of stem cells with autologous fibrin glue in collagen scaffold Control fibrin glue with collagen scaffold alone Control no graft materials	Quadruple	/
NCT01389661	I/II	April 2016	Maxillary cyst Bone loss of substance	Autologous jaw bone marrow MSCs (cultured, pre-differentiated in osteogenic matrix)	11	Application of cells cultured in autologous plasma matrix	None	Treatment resulted in increased cyst density by CT and no adverse effects (Redondo et al., 2018)
NCT02859025	I	February 2016	Cleft of alveolar ridge	Autologous buccal fat pad MSCs (cultured on bovine bone mineral)	10	Cells cultured on bovine bone applied with autologous spongy bone and collagen membrane Cells cultured on bovine bone applied with autologous cortical bone Control autologous spongy bone with collagen membrane	None	Cell-therapy groups showed a trend of higher bone formation after 6 months (Khojasteh et al., 2017)
NCT01932164	NA	December 2015	Cleft lip and palate	Autologous MSCs from deciduous dental pulp (isolated, characterized, frozen)	5	Application of stem cells with collagen and hydroxyapatite	None	Bone formation closing the alveolar cleft after 6 months in all patients
**SEARCH TERMS: “OSTEONECROSIS, STEM CELLS”**								
NCT02448121	I/II	Active, not recruiting	Avascular necrosis of bone in sickle cell disease patients	Autologous bone marrow MNCs	Est. 100	Stem cell injection	None	/
NCT01605383	I/II	Active, not recruiting	Avascular necrosis of the femoral head	Autologous bone marrow MSCs (cultured)	Est. 24	Application of cells with allogeneic bone Control standard treatment only	None	/
NCT02065167	II	Active, not recruiting	Avascular necrosis of the femoral head	Autologous bone marrow MSCs (cultured)	26	Stem cell injection	None	/
NCT01700920	II	December 2015	Osteonecrosis of the femoral head	Autologous bone marrow MSCs (cultured)	3	Stem cell injection	None	/
NCT01643655	NA	March 2015	Avascular necrosis of the femoral head	Autologous adipose MSCs	15	Stem cell injection	None	/
NCT01198080	I	June 2013	Osteonecrosis of the femoral head	Autologous CD133 bone marrow cells	10	Stem cell injection	None	Treatment resulted in disease score improvement, reduced joint injuries and pain relief (Emadedin et al., 2019)
NCT01544712	NA	September 2010	Osteonecrosis of the femoral head	Autologous bone marrow aspirate concentrate	50	Bone marrow concentrate injection Control standard treatment only	Double	Cell therapy did not improve stage 3 osteonecrosis (Hauzeur et al., 2018)
NCT00821470	I	September 2008	Osteonecrosis of the femoral head	Autologous bone marrow aspirate	21	Bone marrow injection Control standard treatment only	Triple	/

*Studies were searched in the Clinicaltrials.gov database (July 2019), using combinations of search terms “bone, bone fracture, stem cells, maxilla, alveolar, osteonecrosis.” Registered studies with unknown status and terminated studies were excluded. Cell type designation in the table is according to the information provided for the specific clinical study in the database*.

Despite this growing knowledge, the translation of MSC-based bone regeneration strategies from research studies to clinical use has been slow (Jakob et al., 2012; Grayson et al., 2015). Limited mechanistic understanding of MSC therapeutic actions and MSC fate following transplantation (Dupont et al., 2010; Manassero et al., 2016) has made the requirements for therapeutic preparations, such as optimal cell numbers, cell phenotype, maturity, and mechanical properties of tissue-engineered grafts difficult to define (Jakob et al., 2012; Oryan et al., 2017). Technical challenges and high costs related to manufacturing under good manufacturing practice (GMP) guidelines and procedures for regulatory approval of MSC therapies (e.g., under advanced therapy medicinal products (ATMP) classification) pose additional barriers for clinical translation (Sensebé et al., 2013). According to our recent search in one of the clinical trials databases (www.clinicaltrials.gov, July 2019, combinations of search terms “bone regeneration,” “bone fracture,” “alveolar bone,” “maxilla,” “osteonecrosis,” and “stem cells”), a number of clinical trials (mostly phase I and I/II) employing MSCs to enhance bone regeneration have been registered. However, only for a few the research findings have been published (Table 1).

**Figure 1:**
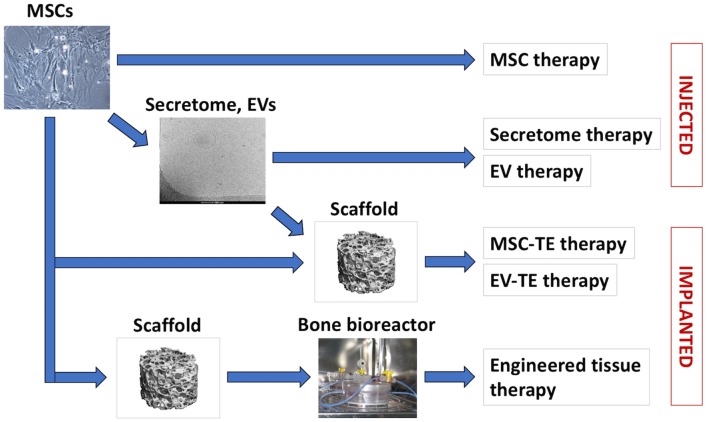
MSC-based bone regeneration strategies. Cultured MSCs can be used for cell therapies or for therapeutic secretome/EVs production (arrow). In tissue engineering (TE) therapies, MSCs or MSC-EVs are applied in combination with biomaterial scaffolds. For certain indications, MSCs are seeded on biomaterial scaffolds and cultured *in vitro* in bioreactors, to support engineered tissue development and maturation prior to application.

In recent years, a shift from the view of MSCs as being cells that directly contribute to new tissue formation toward seeing MSCs as “medicinal cell factories” that secrete a variety of bioactive molecules with trophic and immunomodulatory activities has steered the research into MSC secretome for bone regeneration (Hofer and Tuan, 2016; Caplan, 2017). MSCs also secrete various types of extracellular vesicles (EVs) which contain proteins, lipids, and nucleic acids with potential pro-regenerative properties (Marote et al., 2016; Börger et al., 2017; Turchinovich et al., 2019; van Balkom et al., 2019). Several recent studies in small animal models suggested the therapeutic potential of unfractionated MSC secretome as well as MSC-derived extracellular vesicles for bone regeneration (Table 2). In the current review, we recapitulate the recent developments in bone regeneration strategies employing MSC transplantation and MSC-based tissue engineering, as well as the use of MSC secretome and vesicular fractions (Figure 1). Finally, we discuss the challenges and future perspective of these various MSC-based bone regeneration strategies.

**Table 2:** Preclinical studies reporting the use of MSC-secretome and MSC-EVs for bone regeneration.

**References**	**Cell source**	***In vivo* model**	**Main outcome of secretome treatment**
**UNFRACTIONATED SECRETOME/CONDITIONED MEDIUM**			
Osugi et al. (2012)	Human bone marrow MSCs	Rat calvarial bone defect	Enhanced bone formation (after 4 and 8 weeks), rat MSC migration into the defect
Katagiri et al. (2013)	Human bone marrow MSCs	Rat calvarial bone defect	Early bone regeneration (after 2 and 4 weeks)
Katagiri et al. (2017)	Human bone marrow MSCs	Rat calvarial bone defect	Vascular endothelial growth factor is crucial for angiogenesis and bone regeneration
Ando et al. (2014)	Human bone marrow MSCs Human skin fibroblasts	Mouse distraction osteogenesis model	Accelerated distraction osteogenesis through endogenous cell recruitment of MSC secretome Activity of MSC secretome similar to MSCs transplantation
Kawai et al. (2015)	Human bone marrow MSCs	Rat periodontal defect	Periodontal tissue regeneration (after 4 weeks) and increased presence of CD31, CD105, and Flk1 positive cells
Ogata et al. (2015)	Human bone marrow MSCs	Rat bisphosphonate-related osteonecrosis of the jaw model	Increased bone healing with complete soft tissue coverage; histology demonstrated new bone formation and the presence of osteoclasts
Ogata et al. (2018)	Human bone marrow MSCs Three cytokines mixture	Rat medication-related osteonecrosis of the jaw model	Increased bone healing with soft tissue coverage in conditioned medium and three cytokines mixture groups
Fujio et al. (2017)	Hypoxic human dental pulp cells	Mouse distraction osteogenesis model	Increased blood vessel density and higher bone formation (after 4 weeks)
Xu et al. (2016)	Human fetal bone marrow MSCs	Rat distraction osteogenesis model	Continuous secretome injection improved bone consolidation compared to controls
Wang et al. (2016)	Human fetal MSCs	Mouse ectopic bone formation model	Restored osteogenic capacity of senescent adult human MSCs
**EXTRACELLULAR VESICLES SECRETOME FRACTION**			
Furuta et al. (2016)	Human bone marrow MSCs	CD9 negative mouse fracture healing model	EV injections in the fracture site accelerated fracture healing
Qin et al. (2016)	Human bone marrow MSCs	Rat calvarial bone defect	EV hydrogel application promoted bone regeneration after 8 weeks
Zhang et al. (2016b)	Human ESC-MSCs	Rat osteochondral defect model	Restoration of cartilage and subchondral bone after 12 weeks
Qi et al. (2016)	Human iPSC-MSCs	Ovariectomized rat calvarial bone defect	EV application stimulated bone regeneration and angiogenesis
Li et al. (2018)	Human adipose MSCs	Mouse calvarial bone defect	Enhanced bone regeneration after 6 weeks
Zhang et al. (2019)	Human umbilical cord MSCs	Rat stabilized femoral fracture model	Enhanced angiogenesis and bone healing after 14 and 31 days

## Sources and Culture of MSCs for Bone Regeneration Therapies

According to a consensus position statement, the term MSCs is used to describe a population of multipotent mesenchymal stromal cells that: (1) can be isolated based on their ability to adhere and grow on tissue culture plastic surface, (2) exhibit a defined pattern of positive and negative surface markers, and (3) have the ability to undergo differentiation into osteogenic, chondrogenic, and adipogenic lineages under standard *in vitro* conditions (Dominici et al., 2006). The cells corresponding to this definition were initially isolated from the bone marrow (Friedenstein et al., 1987; Pittenger et al., 1999) and subsequently from many other fetal and adult tissues, including bone, adipose tissue, muscle, blood, dental pulp, placenta, amnion-amniotic fluid, umbilical cord/cord blood etc. (Sudo et al., 2007; Crisan et al., 2008). Importantly, the “stemness” of MSCs, i.e., their ability to self-renew *in vivo*, has only been shown for some MSC subpopulations from the bone marrow by serial transplantation assays (Sacchetti et al., 2007; Méndez-Ferrer et al., 2010). As a surrogate measure of stem cells present in cell preparations, a colony forming units (CFU) assay was commonly used (Robey, 2011) and this was suggested to correspond to the bone forming capacity *in vivo* (Braccini et al., 2007). Only recently, the identity of genuine, self-replicating skeletal stem cells has been described (Chan et al., 2018). Nevertheless, the possibility to relatively easily isolate, culture, and differentiate MSCs from various tissues, particularly those remaining as medical waste (e.g., lipoaspirate, perinatal tissues), has fueled the research into MSCs for bone regeneration therapies.

### Bone Marrow-Derived MSCs

Bone marrow has been investigated in most studies as the standard source of MSCs/progenitors contributing to bone repair *in vivo* (Park et al., 2012; Zhou et al., 2014; Nancarrow-Lei et al., 2017). Bone marrow MSCs have been isolated from individuals of various ages and health backgrounds (Alves et al., 2012; Chadid et al., 2018; Tencerova et al., 2019). Bone marrow MSCs are relatively rare, comprising <0.01% of the isolated mononuclear cell (MNC) fraction (Pittenger et al., 1999). Numbers of isolated MSCs vary between individual patients as well as the site and technique used for tissue harvesting (Muschler et al., 1997; Pierini et al., 2013; Patterson et al., 2017; Herrmann et al., 2019). In order to increase MSC numbers for therapeutic use, protocols for *in vitro* expansion have been developed, employing standardized, animal-supplement-free culture conditions (Schallmoser et al., 2009; Fekete et al., 2012). Bone marrow MSCs can reach over 50 population doublings *in vitro* (Bianco et al., 2001). However, with increased chronological age of the patient and extended *in vitro* culture, bone marrow MSC proliferation and differentiation potentials can decline and the proportion of senescent cells can increase, limiting the therapeutic potential (Stolzing et al., 2008; Churchman et al., 2017; Ganguly et al., 2017).

### Adipose Tissue-Derived MSCs

With the discovery of MSC-like cells in adipose tissue lipoaspirates (Zuk et al., 2001), many studies have turned to this waste tissue as a source for MSC isolation. Volumes of lipoaspirate remaining at plastic surgeries can range from milliliters to several liters and reportedly contain a relatively high proportion of MSCs (between 1 and 5% of the isolated nucleated cells) depending on the donor, harvesting procedure, and tissue harvesting site (Gimble et al., 2007; Jurgens et al., 2008; Dubey et al., 2018). Importantly, some properties of the stromal vascular fraction (SVF) cells isolated from adipose tissue change upon culture *in vitro* (e.g., expression of surface markers) (Gimble et al., 2007), and the cultured cells are subsequently termed adipose MSCs or adipose tissue stromal cells (ASCs) (Bourin et al., 2013). Adipose MSCs exhibit robust osteogenic differentiation potential *in vitro* using standard osteogenic supplements (dexamethasone, beta-glycerophosphate, and ascorbic acid) (Fröhlich et al., 2010; Brennan et al., 2017). However, *in vivo* studies using an ectopic transplantation model in nude mice demonstrated important functional differences between bone marrow and adipose MSCs. When culture-expanded (unprimed) MSCs were transplanted together with osteoinductive calcium phosphate biomaterial or with Matrigel, higher bone ossicle formation and the presence of bone marrow compartment were exclusively found with the bone marrow MSCs as compared to adipose MSCs and cells from other sources (Reinisch et al., 2015; Brennan et al., 2017). This limitation might be overcome by *in vitro* pre-induction of adipose MSCs via the endochondral ossification route (Osinga et al., 2016). On the other hand, adipose MSCs contain vasculogenic subpopulations, which might be an advantage for bone healing by promoting neovascularization (Hutton et al., 2014; Brennan et al., 2017). According to recent reports, regenerative properties of adipose MSCs might not be adversely influenced by age, as is often the case with bone marrow MSCs (Dufrane, 2017; Reumann et al., 2018).

### MSCs Derived From Perinatal Tissues

In addition to bone marrow and adipose tissue, perinatal tissues including umbilical cord, cord blood, amniotic membrane, and placenta are of high interest for bone regenerative therapies, particularly as their collection does not require invasive harvesting procedures (Brown et al., 2019). While autologous use of MSCs from these sources might require cell banking for extended periods, the advantage may be in their younger “chronological” age and thus presumably higher regenerative potential compared to the MSCs from adult/elderly patients. Indeed, umbilical cord MSCs reportedly exhibited a higher proliferation capacity, similar or higher osteogenic differentiation, and absence of adipogenic differentiation as compared to bone marrow and adipose-derived MSCs (Kern et al., 2006; Zhang et al., 2011). Similarly, comparative studies indicated that amnion MSCs have higher proliferation rates and comparable or higher osteogenic differentiation compared to bone marrow and adipose MSCs (Topoluk et al., 2017; Ghasemzadeh et al., 2018), and umbilical cord MSCs exhibited higher proliferation and more rapid osteogenic differentiation compared to bone marrow MSCs (Baksh et al., 2007).

### Induced Pluripotent Stem Cells-Derived MSCs

Populations similar to MSCs have been reported in many other adult and fetal tissues, and evidence of their perivascular location points to their role in responses to injury (Caplan, 2008; Crisan et al., 2008). However, practical aspects, such as quantity of tissue available for harvesting, donor site injury, and limited scientific knowledge might preclude their clinical use. In contrast, practically unlimited numbers of autologous MSC-like cells can be obtained by differentiation of human induced pluripotent stem cells (hiPSCs). hiPSCs are derived from the patient's adult somatic cells by nuclear reprogramming using cocktails of transcription factors (and small molecules) with key roles in the pluripotency regulation network (Takahashi et al., 2007; Zhu et al., 2010; Ma et al., 2013). hiPSCs largely resemble human embryonic stem cells (hESCs) in their pluripotency (i.e., ability to form differentiated tissues of all three germ layers, confirmed *in vivo* by teratoma assay) and differentiation potential (Bock et al., 2011; Bilic and Izpisua Belmonte, 2012). A number of studies reported the differentiation of hiPSCs into MSC-like progenitors (hiPSC-MSCs) and further into bone-like tissue *in vitro* and *in vivo* (reviewed in De Peppo and Marolt, 2013; Luzzani and Miriuka, 2017; Wu et al., 2017). hiPSC-MSCs largely resemble adult MSCs in surface antigen expression pattern, differentiation potential, and global gene expression and thus correspond to the definition of adult MSCs, though variations are observed between individual lines, similarly to adult MSCs (De Peppo et al., 2013). The procedure of their derivation via nuclear reprogramming of adult/aged somatic cells might be used to “rejuvenate” the regenerative potential of cells from elderly patients (Lapasset et al., 2011; Frobel et al., 2014; Spitzhorn et al., 2019). Further investigations into the hiPSC-MSCs phenotype, stability, safety, and *in vivo* development in preclinical models are needed (Jung et al., 2012; Levi et al., 2012; De Peppo et al., 2013; Phillips et al., 2014) prior to their consideration for potential clinical use. Nevertheless, hiPSCs offer a unique opportunity for engineering bone organoids containing various cell lineages from a single unlimited cell source, with broad applications in basic studies and translational applications.

### Heterogeneity and Changes in MSC Properties During *in vitro* Culture

Notably, freshly isolated progenitors as well as cultured MSC populations are highly heterogeneous. Investigations are thus focused on defining the subpopulations endowed with the highest bone regenerative potential that can be procured with minimal manipulation (Caralla et al., 2013; Chung et al., 2015), as well as the development of fast screening methods to predict cell functionality (Li et al., 2016; Murgia et al., 2016). During *in vitro* culture, MSCs progressively lose their proliferation and differentiation potentials and the proportion of senescent cells increases (Stolzing et al., 2008; Churchman et al., 2017). This functional decline can be mitigated to some extent by the adjustment of culture conditions, including specific media supplements (e.g., the widely used basic fibroblast growth factor) (Martin et al., 1997; Chase et al., 2010) and culture on substrates containing extracellular matrix proteins (Mauney et al., 2004, 2006; Chase et al., 2010; Rakian et al., 2015). Such adaptations might not only preserve MSC biological properties, but also allow efficient MSC expansion for cell banking, repeated therapeutic applications, and the preparation of therapeutic secretome.

Taken together, MSCs isolated from different sources exhibit important differences in their availability, characteristics and regenerative potential. Therefore, the choice of cell source and subsequent isolation and manipulation techniques will depend on the requirements of specific research/clinical applications.

## MSCs-Based Therapies and Bone Tissue Engineering

### MSCs in Clinical Applications

Cell therapy approaches to regenerate bone were initially based on the premise that the transplanted MSCs would differentiate and form new bone tissue, thus substituting for the activity of endogenous cells compromised by the injury (Bruder and Fox, 1999; Stegemann et al., 2014; Marcucio et al., 2015). Based on the positive outcomes of preclinical studies (Bruder et al., 1998; Petite et al., 2000; Arinzeh et al., 2003; Granero-Moltó et al., 2009; Caralla et al., 2013; Chung et al., 2015), freshly isolated bone marrow mononuclear cells (MNCs) as well as culture-expanded MSCs from bone marrow, adipose tissue, and dental pulp were evaluated clinically in order to enhance the healing of bone fractures, non-unions, various jaw bone defects, and to prevent bone degradation in femoral head osteonecrosis (Table 1) (Stegemann et al., 2014). For fracture non-unions, fluoroscopy-controlled percutaneous injection of autologous MNCs, concentrated from bone marrow aspirates by centrifugation, i.e., the “Hernigou procedure,” was reported (Hernigou et al., 2005). Successful bone union was obtained in 53 of 60 patients in whom a significantly higher concentration as well as total number of progenitor cells (evaluated by the CFU assay) were transplanted when compared to the 7 patients who did not achieve a bony union. A positive correlation was found between the total number and the concentration of transplanted CFUs and the volume of mineralized callus at 4 months, and a negative correlation was found between the time needed to obtain union and the concentration of CFUs in the graft (Hernigou et al., 2005). Similarly, Le Nail et al. analyzed a series of 43 patients with open tibial fractures with a risk of developing non-unions or presenting non-unions, some of whom received injections of concentrated bone marrow progenitors. They determined a threshold number of transplanted progenitors above which healing was 100% successful (Le Nail et al., 2014).

Quarto et al. first reported the application of culture-expanded bone marrow MSCs together with hydroxyapatite biomaterial for the treatment of large bone defects resulting from traumatic fractures and unsuccessful lengthening in three patients (Quarto et al., 2001). Abundant callus formation along the implants and good integration at the interfaces with the host bones 2 months after surgery were reported. There were no adverse reactions to the implants and all three patients recovered full limb function (Quarto et al., 2001). Several other registered clinical studies used autologous bone marrow MNCs, MSCs, or pre-osteoblasts, either injected or co-applied with bone substitute materials as carriers to treat delayed unions and non-unions of long bones (Table 1). Of these, Emadedin et al. reported that injections of cultured MSCs were safe and evidence of bone union was found in 3 of 5 treated patients (Emadedin et al., 2017). Furthermore, Gomez-Barrena et al. reported that surgical delivery of culture expanded bone marrow MSCs combined with bioceramic granules for the treatment of delayed unions and non-unions was safe and feasible, with 26 of 28 patients exhibiting radiologic healing 1 year after treatment (Gómez-Barrena et al., 2019). For the treatment of tibial, osteoporotic, and mandibular fractures, autologous bone marrow MNCs or MSCs, autologous adipose SVF and autologous or allogeneic adipose MSCs, injected or applied with biomaterials, were studied in comparison to non-cell-therapy controls (Table 1). Liebergall et al. reported that a prophylactic, minimally invasive intervention, involving injection of magnetically-separated bone marrow MSCs, mixed with platelet-rich plasma and demineralized bone matrix (study group, 12 patients), resulted in shorter time to union compared to control group with conventional fracture treatment (12 patients) (Liebergall et al., 2013). Castillo-Cardiel et al. similarly reported that the treatment of mandibular fractures with autologous adipose MSCs (12 patients) resulted in higher ossification rates at 12 weeks compared to the non-cell-therapy control group (12 patients) (Castillo-Cardiel et al., 2017).

Various jaw bone defects were also treated using MSC-therapies, with cells isolated and culture-expanded either from the jaw bone marrow, buccal fat pad, and dental pulp, and subsequently applied in combination with biomaterials (Table 1). Khojasteh et al. reported the treatment of human alveolar cleft defects with buccal fat pad-derived MSCs in combinations with biomaterials. The cell-therapy groups exhibited a trend of higher bone formation after 6 months (Khojasteh et al., 2017). Redondo et al. tested alveolar bone marrow MSCs, osteogenically pre-differentiated within an autologous serum-derived scaffolds *in vitro*, for the treatment of maxillary cysts in 9 patients. They found no adverse effects and an increased density of the cyst interior by computed tomography evaluation (Redondo et al., 2018). Gjerde et al. applied bone marrow MSCs with biphasic calcium phosphate for the treatment of severely atrophied mandibular bone in 13 patients and found no adverse events and sufficient bone regeneration for implant placement after 4–6 months (Gjerde et al., 2018).

Therapies involving autologous bone marrow MNCs or MSCs and adipose MSCs were also evaluated in several studies to treat osteonecrosis of the femoral head (Table 1) (Hernigou and Beaujean, 2002; Hernigou et al., 2018). Hernigou et al. reported that supplementation of the core decompensation procedure with concentrated bone marrow MNCs injections was effective in treating patients with earlier stages of the disease (resulting in less hip replacements), with better outcomes in patients who had greater numbers of progenitors transplanted (Hernigou and Beaujean, 2002). After 20–30 years follow-up, it was reported that core decompression with bone marrow cell injection improved the outcome of the disease (with less hip replacements) as compared with core decompression alone in the same patient group (Hernigou et al., 2018). Recently, injections of magnetically-separated CD133 positive bone marrow progenitors in 9 patients with femoral head osteonecrosis resulted in improved disease scores, less joint injuries, and provided clinically-relevant pain relief (Emadedin et al., 2019). In contrast, Hauzeur et al. reported that implantation of concentrated bone marrow MNCs after core decompression did not produce any improvement in the progression of stage 3 non-traumatic osteonecrosis of the femoral head (Hauzeur et al., 2018).

Taken together, published results of these various clinical studies and reports suggest the overall safety of MSC-based therapeutic approaches, as well as potential enhancement of bone healing compared to control groups. However, differences in clinical indications, study designs and the absence of control groups preclude further mechanistic conclusions. Thus far, no MSC-based therapeutic product has become the standard of care for bone regeneration.

Systemic MSC delivery approaches were also investigated, e.g., for the treatment of osteoporosis (Phetfong et al., 2016), as well as novel therapeutic molecules that would enhance the mobility of endogenous MSCs toward the injured bone sites. In this regard, a biphasic small molecule that recruits osteogenic cells to the bone surfaces was reported to improve bone regeneration in a small animal fracture model (Guan et al., 2012; Yao et al., 2016). It was also reported that co-administration of PTH stimulated systemically administered MSCs to migrate to and regenerate spine injuries and vertebral fractures in preclinical models (Sheyn et al., 2016; Cohn Yakubovich et al., 2017). However, these advances have yet to be implemented and tested in the course of controlled clinical studies to Demonstrate enhanced bone regeneration.

### Bone Tissue Engineering

More “advanced” bone tissue engineering approaches are predominantly in the preclinical research phase. These include various combinations of osteogenic cells, biomaterial scaffolds, signaling factors, and graft culture/maturation procedures (*in vitro* and *in vivo*) toward “functional” bone substitutes. Initially, most *in vitro* studies reported smaller bone constructs (up to several millimeters in size and <0.5 mm thick), since static culture limits new tissue development due to mass transport by diffusion only, as well as batch feeding regimes. Nevertheless, these smaller constructs allowed the evaluation of differences between various bone biomaterials, growth factor delivery regimes, cell differentiation pathways, and cell types to support new bone matrix deposition and mineralization (Meinel et al., 2006; Correia et al., 2012; Marcos-Campos et al., 2012; Chuenjitkuntaworn et al., 2016; Osinga et al., 2016; Rindone et al., 2019). In order to scale-up and standardize these bone tissue engineering strategies to sizes relevant for preclinical studies in large animal models and for clinical applications (beyond reconstruction of smaller jaw bone defects), research has focused on advanced scaffold manufacturing technologies (recently reviewed in Forrestal et al., 2017) and on dynamic tissue culture in bioreactor systems (Meinel et al., 2004, 2005; Marolt et al., 2006; Timmins et al., 2007; Grayson et al., 2008, 2011; Fröhlich et al., 2010; Woloszyk et al., 2014). Perfusion systems that support the interstitial flow of culture medium through bone scaffolds showed the most promise for bone tissue engineering from MSCs originating from adult tissues and pluripotent stem cells (De Peppo et al., 2013; Vetsch et al., 2016; Mitra et al., 2017; Sladkova et al., 2018). The appropriate biochemical milieu and biophysical stimulation provided to the osteogenic cells by the fluid shear force on the cells allowed increased cell numbers and enhanced the uniform cell distribution and the amount of new bone matrix (Sikavitsas et al., 2003; Grayson et al., 2011; Zhao et al., 2018). Grayson and colleagues were the first to report the bioreactor-based engineering of clinically sized, viable human, bone marrow MSCs-derived bone grafts, precisely fitting the complex anatomy of the temporomandibular joint condylar bone (Grayson et al., 2010). In following studies, several centimeter large ramus condyle grafts containing immature bone tissue were engineered from porcine adipose MSCs and evaluated *in vivo* in Yucatan minipigs (Bhumiratana et al., 2016). Six months after implantation, the engineered grafts maintained their anatomical structure, integrated with native tissues and generated greater volume of new bone and greater vascular infiltration than either non-seeded anatomical scaffolds or untreated defects (Bhumiratana et al., 2016). These advances are currently under way to be evaluated in a clinical phase-1 study. Sorensen et al. evaluated the effect of bone marrow MNCs pre-cultured on poly-lactic acid-coated bicalcium phosphate scaffolds in perfusion bioreactors on a spine fusion model in sheep (Sørensen et al., 2012). They found that bioreactor-generated, cell-based bone substitutes were as effective as autologous bone grafts and superior to cell-free bone substitutes in their bone fusion ability. However, bone structure was superior in autografts (Sørensen et al., 2012). In a following study, the bioreactor system was automated for the streamlined production of engineered osteogenic grafts (Ding et al., 2016).

Preclinical studies indicated that the survival of cells in engineered bone grafts can be severely limited after *in vivo* implantation (Giannoni et al., 2010; Becquart et al., 2012; Kaempfen et al., 2015; Manassero et al., 2016). As with autologous grafts, the transplanted tissue that is not immediately connected to the host vasculature is subject to oxygen deprivation and nutrient limitation and the interior portions of the graft undergo necrosis. Pre-vascularization strategies of the tissue-engineered bone grafts are thus an intense area of investigation (Barabaschi et al., 2015). For instance, Güven et al. reported that a 5-days perfusion bioreactor culture of adipose SVF within hydroxyapatite scaffolds resulted in capillary network formation, which anastomosed with the host vasculature already 1 week after ectopic nude rat implantation and promoted a faster tissue ingrowth and more abundant and uniform new bone tissue after 8 weeks, as compared to bone marrow or adipose MSC cultures (Güven et al., 2011). MSCs from different sources might also exhibit different sensitivity to hypoxic conditions. For instance, we previously found that hiPSC-derived MSC engineered bone constructs (~0.5 cm in size) remained viable for 12 weeks in a subcutaneous site, continued to develop, and functional blood vessels were found within the interior portions of the transplants (De Peppo et al., 2013). Furthermore, the application of tissue engineering protocols involving the endochondral differentiation pathway, which is predominant in the healing of long bones, might allow enhanced survival, vascularization, and remodeling of the transplanted hypertrophic cartilage grafts toward new bone regeneration (Bernhard et al., 2017; Epple et al., 2019).

## MSC-Derived Secretome for Bone Regeneration

The native process of bone healing proceeds through overlapping stages of inflammation, repair, and remodeling, which involve multiple signaling pathways acting in concert within the bone defect and the surrounding soft tissues (Oryan et al., 2017). *In vivo* studies examining effects of transplanted MSCs on bone regeneration found limited numbers of transplanted cells surviving and engrafting in the defect sites, and the exact mechanisms of the contribution of exogenous MSCs to new tissue formation are not clear (Geuze et al., 2010; Bhumiratana et al., 2016; Manassero et al., 2016; Oryan et al., 2017). *In vitro* and *in vivo* studies suggested that the transplanted MSCs can have multiple paracrine effects on endogenous cell populations, including immune cell modulation, angiogenic activity, MSC and endothelial progenitor recruitment, cell proliferation, stem cell differentiation, anti-apoptotic effects, and wound healing (Ponte et al., 2007; Chen et al., 2008; Ando et al., 2014; Hofer and Tuan, 2016; Oryan et al., 2017). Depending on their source and manipulation (Oskowitz et al., 2011), MSCs produce a variety of signaling factors, including: purines, bone morphogenetic proteins, CCL2, Connexin 43, cyclooxygenase/prostaglandin, CD95/CD95 ligand, galectins, heme oxygenase-1, human leukocyte antigen-G, interleukin-3, interleukin-6, leukemia inhibitory factor, NO, transforming growth factor beta, vascular endothelial growth factor, hepatocyte growth factor, platelet derived growth factor, basic fibroblast growth factor, and others (Ponte et al., 2007; Chen et al., 2008; Ando et al., 2014; Hofer and Tuan, 2016). The growing understanding of MSC immunomodulatory and trophic activities has steered the research toward the potential of therapeutic MSC secretome, prepared by media conditioning on cultured MSCs, to enhance various stages of bone regeneration (Caplan and Dennis, 2006). In a sense, the use of therapeutic secretome mimics more closely the process of native bone healing by involving multiple signaling factors that work in synergy at low concentrations, rather than a single or a few signaling factors in super-physiologic concentrations (potentially leading to serious side effects, as reported for the high, clinically applied doses of recombinant bone morphogenetic protein therapies) (James et al., 2016). In addition, secretome-based cell-free therapeutic approaches present several significant advantages over current cell- and tissue-based therapies. The absence of replicating (allogeneic) cells in secretome fractions significantly improves the patient safety profile, the low metabolic activity allows for improved quality control and quality assurance, and the simplicity of storage provides the basis for cost-efficient shipping of this potentially off-the-shelf therapeutic substance.

A number of studies evaluated the potential of MSC secretome, either as unfractionated conditioned medium or the extracellular vesicles-enriched fraction, for bone regeneration (Table 2). Secretomes from human adult bone marrow MSCs (Osugi et al., 2012; Katagiri et al., 2013; Ando et al., 2014; Ogata et al., 2015), human dental pulp cells (Fujio et al., 2017), and human fetal MSCs (Wang et al., 2016; Xu et al., 2016) have been investigated. Human adult bone marrow MSC-conditioned media contained insulin-like growth factor-1, vascular endothelial growth factor, hepatocyte growth factor, transforming growth factor beta, monocyte chemoattractant proteins-1 and−3, interleukin-3, and interleukin 6, as determined by ELISA assays (Osugi et al., 2012; Katagiri et al., 2013; Ando et al., 2014; Kawai et al., 2015). Extended analysis of the bone marrow MSC-conditioned medium using a cytokine antibody array yielded 43 proteins with levels at least 1.5-times higher compared to a control medium background (Ando et al., 2014). In particular, serum-free conditioning of the bone marrow MSCs, as reported by most studies (Table 2), was shown to result in a highly angiogenic secretome (Oskowitz et al., 2011).

Osugi et al. reported the potential of human bone marrow MSC-conditioned medium to enhance bone healing in a rat calvarial defect model (Osugi et al., 2012). *In vitro*, this study demonstrated that conditioned medium from human bone marrow MSCs enhanced the migration, proliferation, and expression of osteogenic marker genes, such as osteocalcin and *RUNX2*, in rat bone marrow MSCs (Osugi et al., 2012). Application of the conditioned medium with agarose gel in calvarial defects resulted in higher bone regeneration compared to the agarose gel mixed with human bone marrow MSCs or vehicle controls (phosphate buffered saline or culture medium) after 4 and 8 weeks, presumably by enhancing rat MSC migration into the defect (Osugi et al., 2012). A related study by Katagiri et al. confirmed the *in vitro* findings of Osugi et al. and found that the conditioned medium soaked onto collagen sponges significantly increased early bone regeneration in calvarial defects (after 2 and 4 weeks) compared to the control group (Katagiri et al., 2013). A later study of angiogenesis in the newly regenerated bone suggested vascular endothelial growth factor to be the crucial component in the conditioned medium, promoting angiogenesis and migration of endogenous stem cells (Katagiri et al., 2017). Together, these studies suggested that bone marrow MSC-conditioned medium promotes angiogenesis and bone regeneration in a rat calvarial defect model, but also has the potential to mobilize endogenous MSCs.

Ando et al. demonstrated that repeated applications of human bone marrow MSC-conditioned medium accelerated callus formation in a high-speed mouse distraction osteogenesis model through multiple regenerative mechanisms, similar to the transplantation of bone marrow MSCs (Ando et al., 2014). Analysis of conditioned medium identified factors that recruit mouse bone MSCs, endothelial cells, and endothelial progenitors, inhibit inflammation and apoptosis, and promote osteoblast differentiation, angiogenesis, and cell proliferation. In particular, conditioned medium depleted of monocyte chemoattractant proteins-1 and−3 failed to recruit mouse bone marrow MSCs and callus formation (Ando et al., 2014). Further studies with human bone marrow MSC-conditioned medium showed the potential to enhance regeneration of rat periodontal defects and rat bisphosphonate-related osteonecrosis of the jaw (Kawai et al., 2015; Ogata et al., 2015). In periodontal defects, CD31, CD105, and Flk1 positive progenitors occurred more frequently in the conditioned medium group than controls after 2 weeks and regenerated periodontal tissue was found 4 weeks after implantation (Kawai et al., 2015). In the bisphosphonate-related osteonecrosis of the jaw model, conditioned medium resulted in the healing of open alveolar bone sockets in 63% of the rats (with complete soft tissue coverage and socket bones), compared to the exposed necrotic bone with inflamed soft tissue remaining in the control group (Ogata et al., 2015). Histological analyses demonstrated new bone formation and appearance of osteoclasts with conditioned medium treatment, which was significantly higher compared to the non-treatment group, thus indicating that anti-apoptotic and anti-inflammatory effects of conditioned medium regulated the turnover of local bone (Ogata et al., 2015). A related *in vitro* study indicated that bone marrow MSCs release paracrine factors which directed osteo/odontogenic differentiation of dental pulp cells (Al-Sharabi et al., 2014). Further *in vitro* studies indicated that bone marrow MSC-conditioned medium promoted osteoclast differentiation and expression of master regulatory transcriptional factors for osteoclastogenesis, as well as showed maintenance of osteoclasts despite the presence of RANKL inhibitors (Ogata et al., 2017). Interestingly, a cytokine mixture composed of recombinant monocyte chemoattractant proteins-1, vascular endothelial growth factor, and insulin-like growth factor-1 in concentrations similar to those found in bone marrow MSC conditioned medium, promoted migration, proliferation, and osteogenic differentiation of rat MSCs *in vitro* similarly to conditioned medium, and intravenous application improved the healing of medication-related osteonecrosis of the jaw in a rat model (Ogata et al., 2018).

Fujio et al. studied conditioned media from human dental pulp cells cultured in normoxic and hypoxic conditions (Fujio et al., 2017). Significantly higher angiogenic potential of conditioned media from hypoxic compared to normoxic cultures and no enhancement of either conditioned media compared to controls on the mineralization of human fetal osteoblasts were found *in vitro*. In a mouse distraction osteogenesis model, repeated injections of conditioned medium resulted in increased blood vessel density and higher bone formation compared to the medium control group at 4 weeks (Fujio et al., 2017). In a comparative study of conditioned media from human fetal MSCs, human adult MSCs and rat MSCs, human fetal MSC-conditioned medium showed the highest osteogenic capacity and the lowest immunogenicity *in vitro*, as well as enhanced bone consolidation after repeated injections in the rat distraction osteogenesis model (Xu et al., 2016). A further *in vitro* study reported that the secretome of human fetal MSCs ameliorated replicative senescence and enhanced cell proliferation and the osteogenic differentiation potential of human adult MSCs (Wang et al., 2016). Concomitant activation of *SIRT1* and *FOXO3a* expression, upregulation of p21 gene expression, and downregulation of *BAX* and p53 gene expression were found, and the pre-treatment resulted in restored osteogenic ability of senescent human adult MSCs in a nude mouse ectopic bone formation model (Wang et al., 2016).

Based on the positive outcomes of studies in bone regeneration models, Katagiri et al. reported a first-in-human study and clinical case reports of alveolar bone regeneration using conditioned medium from human bone marrow MSCs (Katagiri et al., 2016). Human bone marrow MSC-conditioned medium was soaked on beta-tricalcium phosphate or on atelocollagen sponge and 8 patients with severe alveolar bone atrophy were treated prior to, or at the same time as dental implant placement. The patients experienced no systemic or local complications and showed early mineralization in the augmented bone according to radiographic evaluations. Calcium phosphate biomaterial structures gradually became indistinct from the surrounding bone 6 months after the surgeries, and biopsies confirmed new bone replacement of the resorbed biomaterial (Katagiri et al., 2016).

## MSC-Secreted Extracellular Vesicles for Bone Regeneration

Growing understanding of MSC intercellular communication via their secretome offers new options for tissue engineering strategies in bone regeneration (Lamichhane et al., 2015). Extracellular vesicles (EVs) are a heterogeneous group of small, lipid-bilayer enclosed, cell-derived particles, exerting effects on fundamental cellular processes in a pleiotropic manner (El Andaloussi et al., 2013). This mechanism is evolutionary conserved from bacteria to humans and plants (Schuh et al., 2019) and was initially considered a means of eliminating unneeded cellular compounds (Johnstone et al., 1987). However, in 1996, Raposo et al. demonstrated that EVs could modulate an adaptive immune response (Raposo et al., 1996) and research from the last two decades has shown that they facilitate intercellular communication, acting as mobile signaling platforms modulating fundamental biological processes in health and disease (Isola and Chen, 2016). Virtually every cell type secretes EVs and they act by the horizontal transfer of proteins, lipids, mRNAs, miRNAs, and other non-coding RNAs, altering the activity of a neighboring or distant target cell (Van Niel et al., 2018). Thereby, the lipid bilayer protects nucleic acids and proteins from degradation in the extracellular environment, allowing their efficient transport. Taken together, these findings have spurred a tremendous amount of effort to exploit EVs as potential therapeutics for immune response modulation and tissue regeneration.

Currently, the term “extracellular vesicles” refers to all types of secreted vesicles released by different cell types under different conditions (Araldi et al., 2012). Based on their biogenesis, two main categories of EVs can be envisioned for therapeutic use: exosomes and microvesicles. Exosomes are rather homogeneous in size (~40–100 nm) and are generated in the endosome as intraluminal vesicles which subsequently mature to multivesicular endosomes. Cargo is sorted into exosomes by distinct mechanisms resulting in the formation of heterogeneous populations of intraluminal vesicles (Colombo et al., 2014). Sorting either involves ESCRT (endosomal sorting complex required for transport), Alix (or PDCD6IP; programmed cell death 6 interacting protein), and TSG101 (tumor susceptibility gene 101 protein) or takes place via an ESCRT-independent mechanism (reviewed in Van Niel et al., 2018). Finally, exosomes are released into the extracellular space upon fusion of the multivesicular endosomes with the cellular plasma membrane. Some of the commonly used markers to identify exosomes are various tetraspanins (CD63, CD81, and CD9), ALIX, TSG101 (tumor susceptibility gene 101 protein), and flotilin-1. However, a recent study demonstrated that classical tetraspanin-enriched exosomes contain a much more limited repertoire of active molecules than has previously been assumed (Jeppesen et al., 2019), highlighting the necessity of suitable protocols for exosome characterization.

Microvesicles (often also referred to as ectosomes or shedding vesicles) represent a rather heterogeneous population of EVs generated by outward budding and subsequent fission of the plasma membrane. Both cargo sorting and subsequent Ca^2+^-dependent vesicle shedding are regulated by various small GTPases, including members of the ARF (ARF6 and ARF1), Rab20, and Rho (Rac1 and RhoA) families (Tricarico et al., 2017). Microvesicles can range in size from ~100 nm to ~1 μm, are rich in phosphatidylserine and are often characterized by the presence of integrins, selectins, and CD40 ligand. Recently, annexin A1 has been proposed as a specific marker for microvesicles which are shed directly from the plasma membrane (Jeppesen et al., 2019). Once EVs are released into the extracellular environment, uptake into target cells takes place either by a receptor-mediated process, internalization by endocytic uptake, or by simple fusion of the cell and vesicle lipid bilayer. However, the unequivocal classification of EVs remains difficult, as the different types share overlapping characteristics and no unique markers are available. Further, current protocols used to purify EVs result in a heterogeneous vesicle population (Willms et al., 2016) and cannot fully exclude a soluble fraction within the purified product, which is why the term “vesicular secretome fraction” has been coined, encompassing both soluble and vesicular components (Gimona et al., 2017).

Several groups provided evidence that EVs are important regulators of stromal cell maintenance (Ratajczak et al., 2006; Quesenberry et al., 2015) and function (Weilner et al., 2016) and it is now widely accepted that much of the efficacy of stromal cell therapies comes from EVs and/or soluble factors (Caplan and Dennis, 2006; Caplan and Correa, 2011). In particular, EVs secreted by MSCs are considered promising candidates for future cell-free regenerative therapies. The paracrine action of MSCs was shown by Gnecchi et al., demonstrating that application of MSC-conditioned medium ameliorated tissue damage in a rodent model of acute myocardial infarction (Gnecchi et al., 2005). Later studies confirmed these results in both pig and mouse models using fractionated MSC secretome preparations (Timmers et al., 2008; Arslan et al., 2013). Since then, the regenerative and immunomodulatory capacity of MSC-derived EVs have been evaluated in several animal disease models, including e.g., kidney and liver injury, lung disease, cartilage repair, hind limb ischemia, ischemic brain injury, and spinal cord injury (reviewed in Harrell et al., 2019).

As MSCs secrete large amounts of EVs (Yeo et al., 2013), EV-based approaches to boost bone regeneration were soon evaluated. As pointed out earlier, in comparison with direct MSC transplantation, MSC-derived EVs appear *prima facie* safer, as they are devoid of viable cells. Further, systemically applied EV preparations are less likely to be trapped in the lung or liver and are most likely less immunogenic (Lai et al., 2013). Finally, they can be stored for an extended period of time, offering the possibility of an off-the-shelf product for restoration of bone defects (Webber and Clayton, 2013). Indeed, EVs purified from bone marrow MSCs, umbilical cord MSCs, endothelial progenitor cells, and iPSC-MSCs enhanced healing of bone in rodent models (Furuta et al., 2016; Qi et al., 2016; Jia et al., 2019; Zhang et al., 2019). Furuta et al. isolated EVs from human bone marrow MSC-conditioned medium and injected it into femoral fractures in a CD9 negative mouse strain (which produces low levels of EVs) and wild type mice (Furuta et al., 2016). Preparations of EVs contained low levels of bone-repair related cytokines. However, their application accelerated fracture healing compared to control (vehicle treated) animals (Furuta et al., 2016). Repeated injections of EVs prepared from human ESC-MSCs similarly promoted the regeneration of osteochondral defects in a rat femur model (Zhang et al., 2016b) and enhanced bone regeneration was found in a rat distraction osteogenesis model with injections of EVs prepared from rat endothelial progenitors (Jia et al., 2019).

More recently, MSC-EVs combined with various scaffold materials generated bone *in vivo* in ectopic sites (Xie et al., 2017) and successfully promoted bone repair in rodent calvarial bone defects (Qi et al., 2016; Qin et al., 2016; Li et al., 2018). Ideally, biocompatible scaffolds should degrade at an appropriate rate and facilitate the controlled release of the extracellular vesicles, as mere loading results in a burst release and might be less efficient in exerting pro-regenerative effects. Qi et al. showed in a calvarial defect model, established in osteoporotic rats, that treatment with tricalcium phosphate scaffolds loaded with EVs prepared from hiPSC-MSCs repaired bone defects through enhanced angiogenesis and osteogenesis (Qi et al., 2016). More recently, Li et al. reported that human adipose MSC-derived EVs immobilized onto poly-lactic-co-glycolic acid scaffolds exhibited a slow release profile *in vitro* and enhanced bone regeneration in mouse calvarial defects after 6 weeks (Li et al., 2018).

However, the molecular and cellular mechanisms underpinning the osteogenic effect of EVs remain poorly defined and they are certainly cell- and tissue context-dependent (Harrell et al., 2019). Potentially, they can be attributed to a protective effect in necrotic and ischemic environments (Liu et al., 2017), recruitment of endogenous MSCs (Osugi et al., 2012; Furuta et al., 2016), and HIF-1alpha-dependent pro-angiogenic activity (Qi et al., 2016; Zhang et al., 2019). In addition, MSC-EVs seem to directly promote osteogenic differentiation, in part by activating the PI3K/AKT signaling pathway (Zhang et al., 2016a) and via the activity of miRNA-196a released from the EVs (Qin et al., 2016). In general, MSC-derived extracellular vesicles have been shown to exert anti-inflammatory properties by inducing high levels of anti-inflammatory proteins, while concomitantly attenuating pro-inflammatory cytokines in THP-1 monocytes *in vitro* and inducing regulatory T cells *in vivo* (Zhang et al., 2014). Taken together, MSC-EVs elicit pleiotropic effects, promoting bone repair. However, it is important to note that the characteristics of the microenvironment influence the function of EVs produced by these cells (Huang and Feng, 2017). Along these lines, Zhu et al. demonstrated that the pro-angiogenic and osteogenic actions of bone marrow MSC-EVs are impaired in type-1 diabetes (Zhu et al., 2019). Therefore, it is imperative to choose appropriate cell sources for EV production for future therapeutic strategies. Taken together, EV-based, cell-free therapies appear to be a promising strategy to repair bone tissue. However, to ensure comparability between preclinical studies and to allow future translation into the clinic, EV purification and characterization protocols urgently need to be harmonized (Witwer et al., 2019).

## Production of MSCs, Secretome, and Extracellular Vesicles for Clinical Use

Production of MSCs for clinical use requires appropriate laboratory procedures adhering to GMP regulations. These procedures have been reported previously by several groups for bone marrow MSCs (Schallmoser et al., 2009; Fekete et al., 2012). Similarly, the manufacturing of MSC-derived therapeutic secretome and EVs for clinical testing requires GMP-compliant strategies, and several groups focused on establishing such protocols (Andriolo et al., 2018; Mendt et al., 2018; Rohde et al., 2019). Existing GMP protocols for MSC expansion can be largely adopted for the first step in secretome/EV production and standard operating procedures can be expanded by the concomitant processing steps of the conditioned medium. Overall, the therapeutic secretome/EV field benefits at this point from the experience gained from MSC manufacturing for clinical applications as well as from the ample experience available from virus-like particle enrichment. In an excellent overview on the current status of EV manufacturing, Whitford and Guterstam (2019) indicated that the technologies and knowledge for GMP-compliant EV production are at hand. However, some adaptation and fine tuning is still required. For instance, it should be noted that large scale production of therapeutic MSCs in order to obtain significant batch sizes of either cells or EVs for preclinical and clinical testing may alter the physiological state of the MSCs. The influence of cell passage number on the therapeutic potential of MSC-derived EVs has been questioned at least for adipose MSCs (Serra et al., 2018). Furthermore, the use of serum-free medium can cause changes in exosome biology and cargo sorting, and the use of coatings (e.g., fibronectin, gelatin) alters the profile of MSC adhesion and mechanotransduction and automatically violates a central requirement for defining MSCs, namely plastic adherence (Brindley et al., 2011; Whitford and Guterstam, 2019).

Bioreactor systems and associated analytical approaches have been studied for scale-up and a more efficient, reproducible, safe and cost-effective production of MSCs and secretome compared to static culture (Carmelo et al., 2015; Mizukami et al., 2019). Carmelo et al. developed a xeno-free microcarrier-based stirred culture system for scalable expansion of bone marrow and adipose MSCs. Their secretome analysis suggested a priming effect of stirred culture conditions toward potentially increased production of specific cytokines by MSCs (Carmelo et al., 2015). Similarly, a study by Teixeira et al. indicated that culture of bone marrow MSCs on microcarriers using computer-controlled suspension bioreactors enhanced the neuroregulatory profile of the secretome compared to static cultures (Teixeira et al., 2016). Secretory profiles of the MSCs can also be modulated by the surface structure of the microenvironment (Leuning et al., 2018), pointing further to the enhanced potential of engineered three-dimensional culture environment for optimizing MSC-derived therapeutic products.

## Future Directions and Challenges for MSC-Based Bone Regeneration Approaches

Within the field of bone regeneration, there is a clear need for new cell-based or cell-free therapies for a number of conditions in which bone does not heal, resulting is significant morbidity and burden for the patients. At this time, several MSC-based therapies have reached the stage of clinical trials, but none of the approaches has been accepted in the clinic as the standard of care (Stegemann et al., 2014). Additional research is needed to advance our mechanistic understanding of various cell-, EV-, and tissue engineering-based therapies within specific clinical indications.

For MSC-based cell therapies, some of the currently registered clinical studies are starting to address the questions of dosing, the use of autologous vs. allogeneic cells, and efficacy compared to standard treatments using autologous bone grafts. However, many challenges remain in the standardization, quality control, and potency evaluation of MSC-based cell therapies, as well as in the scale-up, GMP manufacturing, and logistics, in particular when autologous MSCs are used. In addition to inter-patient variability due to age, health, and various risk factors, inherent differences between MSCs from various tissue sources present specific advantages and disadvantages, such as the potential to be harvested in high or low yields, surgical procedures and donor site morbidity, potential of use with minimal intra-surgical manipulation/preparation of the cells, the need for additional cell storage and culture of the cells to increase their numbers, co-application of signals to promote cell phenotype/differentiation, local vs. systemic delivery via injections or in combination with scaffolds (i.e., tissue-engineered therapies). For certain indications, e.g., complex facial bone defects, extended *in vitro* or *in vivo* engineering of viable bone substitutes might be required to provide appropriate architecture as well as mechanical properties for defect stabilization and loading, which are key for successful healing. For others, MSC injections or co-application with bone substitute materials might provide the critical components required for successful outcomes.

Due to these complexities with viable cells and tissue transplantation, a number of investigations have also focused on enhanced mobilization and homing of endogenous cells (which has not been extensively investigated in the clinic) (Yao and Lane, 2015; Karlsson et al., 2016; Yao et al., 2016) and on novel, secretome-based therapeutic approaches. Whereas clinical translation of the whole secretome might prove challenging as well, re-constitution of major secreted growth factor components (e.g., by using recombinant proteins) in concentrations similar to those in the MSC secretome might recapitulate the therapeutic effects, as was recently demonstrated (Ogata et al., 2018). Of note, repeated applications of conditioned media were used in several studies to achieve the therapeutic effects, in contrast to single applications in the case of cell- and tissue-engineering-based therapeutic approaches. Surviving transplanted cells/tissues will be exposed to the effects of the local environment in the defect and might change their functions as a result. In contrast, preparation of therapeutic secretome might incorporate *in vitro* culture modifications to modulate the composition of released components.

Therapies based on EVs face similar problems as cell therapies in translating the promising research data into well-defined clinical trials and subsequent applications to patients. A central issue in the field is the standardization of manufacturing and analytic processes. A prerequisite for this advancement seems to be the awareness that any cell-derived EV product will always comprise of a heterogeneous mixture of extracellular vesicles and in most cases also of co-purifying soluble components. While this heterogeneity may be more of a concern for naive, unmanipulated MSC-EV preparations, loading and engineering of EV products to contain specific signals (Sutaria et al., 2017) might be less affected, with a greater focus on the characterization of the active component (e.g., siRNAs or engineered proteins) and its therapeutic potential.

Intuitively, the discussion on the best cell source for use in bone regeneration will always consider that cells that perform better in *in vitro* differentiation assays toward osteogenic lineage may be better suited for the manufacturing of EVs that support or enhance bone healing. It must be emphasized though, that these assays are highly artificial and the link between *in vitro* cell differentiation potential and the regenerative potential of cell-derived EVs must be further evaluated. Current efforts in the EV community are focused on determining the best producer cell source, on potential modes of action, and on standardization of physico-chemical assays and analytical devices for functional EV characterization. At the present time, analytical methods that are specifically tailored to characterize therapeutic EVs are largely missing. Nanoparticle tracking analysis is capable of determining the total number of particles in the heterogeneous (secretome) solution, but neither the particle size nor the particle number can currently predict the functionality of the product. Cell-based potency assays are difficult to establish to the level of GMP compliance and will have to be designed specifically for each clinical indication.

There is also no current consensus on the best method to expand the producer MSCs (two-dimensional vs. three-dimensional culture, batch-feed vs. stirred tank bioreactor, serum supplemented vs. serum free/defined medium) or to enrich/purify EVs or secretome. Therefore, application for clinical trials cannot rely on or reference to already approved investigational new drug/investigational medicinal products. This is well-reflected by the mere four interventional studies with EVs that are listed at clinicaltrials.gov. To some, the current classification of EV-containing therapeutic substances as biologicals by both EMA and FDA seems to be an advantage, since ATMP regulations do not apply. It remains to be seen, however, how the regulatory agencies deal with the application on a case-by-case basis in the absence of clear and binding regulations.

Finally, prospective manufacturers of EV therapeutics have to take into consideration that EV-based products for improved bone healing and regeneration aim, for the most part, at non-life-threatening medical indications. Irrespective of the considerable benefit for the patient and the health care system, this fact impacts the possible pricing of the product and thus the total manufacturing cost. For instance, the current financial requirement for available CAR-T cell therapy (430,000$ US) is an extreme example of the costs of cell-based therapy. Therapies based on EVs toward bone healing should look more at costs in the range of 1,000–2,000$ US to make the treatment both attractive and suitable for refund by the health insurance providers. This, however, will again demand precise calculations of the cost of goods and manufacturing costs, as well as for costs for testing, release, and storage. In this respect, batch size and dosing once again take center stage and must be considered in the development of a GMP-compliant manufacturing process for EV therapeutics and EV-enriched secretome.

In conclusion, further advancements in the science and translation of MSC-based therapies are required, and this growing body of knowledge, in conjunction with the improved identification of clinical indications and patients best suited for these treatments, will be key to creating therapies that will be consistently more successful than current treatments, while also being cost effective and marketable.

## Author Contributions

All authors listed have made a substantial, direct and intellectual contribution to the work, and approved it for publication.

### Conflict of Interest

The authors declare that the research was conducted in the absence of any commercial or financial relationships that could be construed as a potential conflict of interest.

## Abbreviations

ASCs: adipose stromal cells
ATMP: advanced therapy medicinal product
CFU: colony forming units
EV: extracellular vesicle
GMP: good manufacturing practice
hESCs: human embryonic stem cells
hiPSCs: human induced pluripotent stem cells
MNCs: mononuclear cells
MSCs: mesenchymal stromal cells
NO: nitric oxide
SVF: stromal vascular fraction
TE: tissue engineering.
